# Bisphenol E Neurotoxicity in Zebrafish Larvae: Effects and Underlying Mechanisms

**DOI:** 10.3390/biology14080992

**Published:** 2025-08-04

**Authors:** Kaicheng Gu, Lindong Yang, Yi Jiang, Zhiqiang Wang, Jiannan Chen

**Affiliations:** 1 School of life Science, Nanjing Normal University, No. 1 Wenyuan Road, Qixia District, Nanjing 210023, China; 18362159300@139.com; 2 Department of Obstetrics and Gynecology, School of Medicine, Nanjing University, 305 Zhongshan East Road, Xuanwu District, Nanjing 210018, China; yanglingdong-123@163.com; 3 Department of Animal Science, College of Animal Science, Hebei North University, Zhangjiakou 075000, China; 15996209564@163.com; 4 Department of Anorectal Surgery, Tianjin Medical University, No. 23 Pingjiang Road, Hexi District, Tianjin 300211, China; 5 Jiangsu Key Laboratory for Molecular and Medical Biotechnology, College of Life Sciences Nanjing Normal University, 1 WenYuan Road, Nanjing 210023, China

**Keywords:** endocrine-disrupting chemicals, bisphenol E, neurotoxicity, network toxicology

## Abstract

As typical environmental hormones, endocrine-disrupting chemicals (EDCs) have become a global environmental health issue of high concern due to their property of interfering with the endocrine systems of organisms. As a commonly used substitute for bisphenol A (BPA), bisphenol E (BPE) has been frequently detected in environmental matrices such as soil and water in recent years. Existing research has unveiled the developmental and reproductive toxicity of BPE; however, only one in vitro cellular experiment has preliminarily indicated potential neurotoxic risks, with its underlying mechanisms remaining largely unelucidated in the current literature. Potential toxic mechanisms and action targets of BPE were predicted using the zebrafish model via network toxicology and molecular docking, with RT-qPCRs being simultaneously applied to uncover neurotoxic effects and associated mechanisms of BPE. A significant decrease (*p* < 0.05) in the frequency of embryonic spontaneous movements was observed in zebrafish at exposure concentrations ≥ 0.01 mg/L. At 72 hpf and 144 hpf, the larval body length began to shorten significantly from 0.1 mg/L to 1 mg/L, respectively (*p* < 0.01), accompanied by a reduced neuronal fluorescence intensity and a shortened neural axon length (*p* < 0.01). By 144 hpf, the motor behavior in zebrafish larvae was inhibited. Through network toxicology and molecular docking, HSP90AB1 was identified as the core target, with the cGMP/PKG signaling pathway determined to be the primary route through which BPE induces neurotoxicity in zebrafish larvae. BPE induces neuronal apoptosis and disrupts neurodevelopment by inhibiting the cGMP/PKG signaling pathway, ultimately suppressing the larval motor behavior. To further validate the experimental outcomes, we measured the expression levels of genes associated with neurodevelopment (*elavl3*, *mbp*, *gap43*, *syn2a*), serotonergic synaptic signaling (*5-ht1ar*, *5-ht2ar*), the cGMP/PKG pathway (*nos3*), and apoptosis (*caspase-3*, *caspase-9*). These results offer crucial theoretical underpinnings for evaluating the ecological risks of BPE and developing environmental management plans, as well as crucial evidence for a thorough comprehension of the toxic effects and mechanisms of BPE on neurodevelopment in zebrafish larvae.

## Simple Summary

Bisphenol E (BPE), a typical endocrine disruptor, is widely present in various environmental media. Using zebrafish as a model, this study systematically evaluated the toxic effects of BPE on embryonic development and the nervous system. By integrating a network toxicology analysis and molecular docking techniques, the neurotoxic mechanism was revealed: BPE inhibits the activity of NOS3/PKG, blocks the transmission of the cGMP/PKG signaling pathway, and ultimately induces neurotoxic damage. This research advances the understanding of endocrine disruptor toxicity by providing novel experimental evidence and delivers key theoretical pillars for building a health risk assessment system targeting environmental pollutants.

## 1. Introduction

Endocrine-disrupting chemicals (EDCs), also known as environmental hormones, are a class of chemical substances that interfere with the endocrine system functions of organisms. Among them, bisphenols represent one of the most widely studied groups of EDCs. Due to their environmental persistence and bioaccumulative properties, pollution caused by bisphenols has emerged as a significant environmental challenge. For instance, bisphenol A (2,2-Bis(4-hydroxyphenyl)propane, BPA)—recognized for its developmental, neurotoxic, and reproductive hazards—has been progressively banned in several countries [1]. As a typical substitute for BPA, bisphenol E (bis(4-hydroxyphenyl)ethane, BPE) is used as a raw material in the production of polycarbonate plastics and epoxy resins and is widely applied in industrial manufacturing, such as the fabrication of plastic containers, electronic device components, coatings, adhesives, and other polymer materials [2].

With the widespread use of BPE, bisphenol E has now been commonly detected in multiple matrices, including water bodies, food packaging, building materials, and human bodies. For example, traces of BPE have been identified in water environment-related samples, such as source water, drinking water, and bottled water, with concentrations reaching ng/L [3]. Studies indicate that wastewater treatment processes serve as significant accumulation points for BPE. Notably, BPE was first detected in sewage sludge in China [4]. In Xiamen, China, the influent of wastewater treatment plants contained BPE at an average concentration of 4.51 ng/L [5], while in a Slovenian wastewater treatment plant, the maximum concentration in influent water reaches as high as 238 ng/L [6]. In the food sector, low concentrations of BPE have been detected in food products on the Swiss and EU markets. For instance, vegetable soup samples showed a concentration of 1.24 μg/kg; canned tuna samples contained 1.28 μg/kg; and fruit puree samples had concentrations ranging from 0.53 to 1.37 μg/kg [7]. BPE has also been detected in fresh milk from major local and national brands in China, at a concentration of 0.04 ng/mL [8]. In building materials, the concentration of BPE in epoxy resins is 1.04 ± 0.02 mg/L [9]. Of particular concern is human exposure levels: a study examining urine samples from 1054 mother–infant pairs detected BPE in 93.0% of samples, with a median exposure level of 0.214 ng/mL. This high detection rate and exposure level suggest that the potential risks of BPE to the development of human offspring cannot be underestimated [10]. The above research indicates that BPE is commonly detected in various substances closely related to human life, such as water, building materials, and food. Its long-term impacts on ecological environments and human health urgently require further in-depth research and monitoring.

Due to its structural similarity to BPA, BPE is recognized as a potential EDC and has been reported to elicit multiple adverse effects. Studies have shown that BPE exhibits an acute toxicity comparable to the well-known BPA [11]. The 96 h lethal concentration 50 (96h-LC_50_) values for zebrafish embryos exposed to BPE and BPA are 13.61 mg/L and 11.69 mg/L, respectively. Additionally, research indicates that the exposure of Drosophila larvae to 1.62 mM BPE significantly delays developmental progression, directly interfering with normal neurodevelopmental processes [12]. In vitro differentiation models using human embryonic stem cells (hESCs) have demonstrated that exposure to 100 nM BPE drastically shortens both the total length and maximum length of neurites in neuron-like cells, similarly exerting adverse effects on neurodevelopment [13]. However, the specific mechanisms underlying BPE’s impact on neurodevelopment remain insufficiently and incompletely explored.

The exceptional molecular and physiological attributes of zebrafish (*Danio rerio*) underpin their significance as a prominent aquatic model organism in neurotoxicity studies of environmental contaminants. The ex utero development and optical clarity of zebrafish embryos allow the non-invasive real-time visualization of organs/tissues, greatly simplifying the tracking of pollutant-driven physiological responses. Their conserved vertebrate genetics and human-like physiological traits—particularly complex nervous systems—further validate their model relevance [14]. Key investigative priorities for environmental pollutant neurotoxicity in zebrafish encompass morphological traits, behavioral modulation, oxidative stress, gene expression patterns, and neurodevelopmental mechanisms [15]. As such, zebrafish have become an ideal model for studying neurological disorders and toxicological effects, not only providing a powerful tool for identifying and characterizing neurotoxins but also establishing a highly valuable research platform for deepening investigations into the underlying mechanisms of nervous system diseases.

In the current research on pollutant mechanisms, network toxicology and molecular docking have emerged as core methods for deciphering pollutant–biology interaction mechanisms [16]. Network toxicology, an emerging discipline integrating principles of network pharmacology and network biology, facilitates the construction of comprehensive “toxicant signature–compound–gene–protein” models to elucidate the mechanistic characteristics of BPE [16]. Enabling the robust analysis of protein interactomes and the prediction of disease progression mechanisms induced by toxicant exposure, this approach offers an integrative platform [17]. Molecular docking, a computational structure-driven method, enables the prediction of interactions between ligands and targets at the molecular level and the delineation of structure–activity relationships without prior knowledge of other target modulators’ chemical structures [18]. When applied to toxicological research, molecular docking can predict and clarify interactions between toxins and biomolecules, uncovering toxic mechanisms and potential hazards these substances may pose to biological organisms [19]. Integrating network toxicology with molecular docking, this study predicted BPE toxicity to establish a theoretical foundation for exploring its neurotoxic mechanisms in zebrafish larvae.

This study employs zebrafish models to mechanistically dissect the neurotoxic effects of BPE using an integrated approach combining network toxicology, molecular docking, and a quantitative real-time PCR (RT-qPCR) aimed at identifying molecular targets and associated pathways. As a potential EDC, elucidating BPE’s mechanisms provides critical scientific evidence to inform product safety, environmental risk management, and the protection of both ecological systems and human health.

## 2. Materials and Methods

### 2.1. Chemical Substances

4,4′-Ethylidenebisphenol (BPE, CAS No.: 2081-08-5) was purchased from Shanghai Aladdin Biochemical Technology Co., Ltd. (Shanghai, China). Dimethyl sulfoxide (DMSO) (CAS: 67-68-5) was purchased from Beijing Solarbio Science & Technology Co., Ltd. (Beijing, China). All chemical reagents and solvents employed in the investigation were of analytical grade.

### 2.2. Zebrafish Cultivation and Maintenance

The transgenic strain *Tg(huc:eGFP)* with a fluorescently labeled central nervous system and the wild-type AB strain of zebrafish (*Danio rerio*) were acquired from the Chinese Academy of Sciences’ Institute of Hydrobiology (Wuhan, China). All sexually mature adult fish were reared in an intelligent recirculating aquaculture system equipped with a three-stage physical–biological filtration unit and an ultraviolet disinfection module. The water temperature was strictly maintained within the physiologically optimal range of 26.0 ± 0.5 °C via temperature control devices, and the light cycle was set to 14L:10D (light onset at 08:00) by a timed program controller, simulating the photoperiod rhythm of the native habitat. Nutrient-rich active brine shrimp were fed twice daily at 09:00 and 16:00. Prior to breeding experiments, healthy broodstock with well-developed gonads were selected and placed in breeding tanks with removable partitions at 20:00 on the breeding day at a male-to-female ratio of 1:2. The bottom of the tank was covered with a sieve to prevent the broodstock from eating the eggs. The partition was removed at 08:00 the following day, and natural spawning was completed within 30 min. The collected embryos were immediately rinsed three times with aerated water. Morphological screening was performed under a Nikon SMZ18 stereomicroscope (Nikon, Tokyo, Japan), and embryos that met the criteria were excluded for subsequent experiments. All animal procedures were conducted in accordance with the provided Guidelines for the Care and Use of Laboratory Animals (*IACUC-20250401*) (Figure S1).

### 2.3. Acute Toxicity Test and General Developmental Toxicity Test of BPE

One milligram of BPE was weighed, and a stock solution of BPE with a concentration of 1 × 10^3^ mg/L was prepared using 100 μL of DMSO and 900 μL of water. The solution was homogenized by shaking and sonication, after which serial dilution was performed to prepare the required concentrations. Ten wild-type zebrafish embryos were placed in each well of a 6-well plate and exposed to BPE at concentrations of 0, 10, 20, 40, and 80 mg/L until 96 hpf. Embryos were monitored three times a day to record and eliminate dead embryos, and exposure solutions were changed every day. Each treatment was repeated in triplicate, and the calculated LC_50_ was 17.72 mg/L, which was consistent with existing experimental results [11]. Based on the acute toxicity test results and environmental concentrations [9], BPE concentrations were set at 0, 0.01, 0.1, and 1 mg/L. In the following tests, 30 transgenic zebrafish embryos in each dish were put in Petri dishes with 20 mL exposure solution, while 10 wild-type zebrafish embryos per well were assigned to plates with 5 mL of aerated water. Transgenic embryos were used for microscopic examination in both settings, which kept three duplicates per concentration. During the experiment, survival rate and hatching rate were recorded every 24 h using a fluorescent stereomicroscope. At 24 hpf, photographs were taken to count the frequency of embryonic spontaneous movements; at 72 hpf, heart rate and body length were measured; and at 144 hpf, body length was recorded (*n* = 12).

### 2.4. Behavioral Testing of Zebrafish Larvae

Locomotor activity of 6 dpf zebrafish larvae was assessed under alternating light–dark cycles. Twelve larvae per treatment group were individually transferred to 24-well plates containing 3 mL test water. After 10 min acclimation (26 ± 0.5 °C), activity was recorded during 40 min alternating light–dark cycles (10 min light: 10 min dark). Motor behavior was continuously recorded using the DanioVision behavioral observation system (Noldus, Wageningen, The Netherlands), and the accompanying EthoVision XT 15 animal movement trajectory analysis software was employed to calculate and statistically analyze the distance swum and movement speed under light and dark conditions. Each experiment was repeated three times (*n* = 12).

### 2.5. Studies on the Neurodevelopmental Toxicity of Zebrafish Larvae

Given observed BPE-induced motor deficits, transgenic *Tg(huc:eGFP)* zebrafish were exposed to graded BPE concentrations to assess neuronal developmental impacts. Per treatment group, 12 larvae were fixed in 4% paraformaldehyde (5 min). Fluorescent stereomicroscopy (Nikon SMZ25, Tokyo, Japan) was used to observe larvae exposed to BPE until 72 hpf and 144 hpf, with statistical analysis of fluorescence expression intensity in transgenic *Tg(huc:eGFP)* embryos. The fluorescence intensity of green fluorescent protein in zebrafish larvae was quantitatively analyzed using NIS-Elements D software (version 5.41.00; Nikon, Tokyo, Japan).

### 2.6. Target Identification for BPE-Induced Neural Damage and PPI Network Construction

First, the CAS number “2081-08-5” was input into the PubChem database to verify the accuracy of the compound BPE’s name and molecular formula. Upon confirmation, the SDF (Structure Data File) of its two-dimensional structure was downloaded. This file was then uploaded to the Swiss Target Prediction database (http://www.swisstargetprediction.ch/, accessed on 20 April 2025) [20], where the task was submitted and prediction results were downloaded to obtain the potential target set of BPE. For the construction of a neural damage-related disease target library, reliance was primarily placed on the GeneCards database (http://www.genecards.org/, accessed on 21 April 2025) [21]. Given the focus of this study on nervous system injury, the keyword “neural damage” was input into the database for searching, and relevant target sets were obtained after screening and deduplication. Finally, using the Venn Diagram online tool (http://bioinformatics.psb.ugent.be/webtools/Venn/, accessed on 24 April 2025) [22], the BPE target library and neural damage target library were uploaded separately, and the intersection analysis function was used to submit the computation, ultimately yielding the common target results between the two.

The PPI network was primarily constructed using the STRING database (https://string-db.org/, accessed on 5 May 2025) [23]. Targets overlapping between the BPE and neural damage target libraries, identified via Venn diagram, were uploaded to the STRING database (species: *Homo sapiens*) to generate a PPI network. Subsequently, Cytoscape software (3.10.3) was used to analyze, optimize the network structure, and identify key nodes. Core target screening followed the degree value criterion, where only targets with degree values greater than or equal to twice the median were selected as core research objects. Detailed results are presented in Table S1.

### 2.7. GO and KEGG Pathway Analysis

To decipher the biological processes and signaling pathways involved in BPE’s action on neural damage-related common targets, Gene Ontology (GO) and Kyoto Encyclopedia of Genes and Genomes (KEGG) enrichment analyses were performed using the DAVID database (https://david.ncifcrf.gov/, accessed on 7 May 2024) [24]. The specific procedure was as follows: the list of common targets obtained from the Venn diagram analysis was uploaded to the database, with the species set as *Homo sapiens*, and the computation was submitted after parameter configuration. For result processing: in the KEGG pathway enrichment results, the top 10 key pathways were selected by sorting enrichment significance (*p*-value) in ascending order. For the GO enrichment analysis, the top 10 significantly enriched entries with the smallest *p*-values were, respectively, chosen from the three ontologies of Biological Process, Cellular Component, and Molecular Function, forming a hierarchical analysis result.

### 2.8. Molecular Docking

Molecular docking studies were conducted using a combination of AutoDock-1.5.6 and PyMOL 2.3.4 software, following the procedure below: First, three-dimensional structure files (PDB format) of human (*Homo sapiens*) target proteins (receptors) were retrieved from the RCSB PDB database (https://www.rcsb.org, accessed on 7 May 2024) [25]. Using PyMOL, water molecules, ligand molecules, and irrelevant cofactors were removed from the receptor structures, retaining only the protein backbone, which was saved in PDB format. Second, SDF structure files of the target ligands were obtained from the PubChem database, converted to PDB format using Open Babel, and preprocessed using AutoDock Tools-1.5.6—steps included adding hydrogen atoms, removing crystalline water, and calculating partial atomic charges—with the final ligand files saved in pdbqt format.

During receptor preprocessing, the cleaned receptor PDB files were imported into AutoDock Tools-1.5.6 to remove redundant atoms and generate receptor pdbqt files. In AutoDock-1.5.6, Grid Box parameters (including central coordinates and grid dimensions) were set, and the molecular docking program was run after completing parameter configuration. Upon completion of docking, Discovery Studio and PyMOL 2.3.4 were used collaboratively for visual analysis of docking results and screening of high-affinity conformations in ligand–receptor complexes.

### 2.9. Fluorescence Quantitative PCR in Real Time

Total RNA was isolated from 50 zebrafish embryos per exposure group at 144 hpf using Trizol reagent (Takara, Dalian, China), with three biological replicates per group. cDNA was synthesized using the PrimeScript^®^ RT Kit (TaKaRa, Kyoto, Japan). qPCR was performed on a Bio-Rad CFX Connect system (Bio-Rad, Hercules, CA, USA) with SYBR Green detection (Vazyme Biotech Co., Ltd., Nanjing, China) and primers from Sangon Biotech (Shanghai, China). Expression levels of target genes were quantified, including neurodevelopment-related (*elav3*, *mbp*, *gap43*, *syn2a*), serotoninergic signaling-related (*5-ht1ar*, *5-ht2ar*), cGMP/PKG pathway-related (*nos3*), and apoptosis-related (*caspase-3*, *caspase-9*) genes (see Table S2). β-actin served as the reference gene, and relative RNA levels were calculated using the 2^−ΔΔCt^ method.

### 2.10. Statistical Analysis

Statistical analyses were performed using GraphPad Prism 8 (GraphPad Software, Boston, MA, USA). Significant differences between exposure groups and the control group were denoted as follows: *p* < 0.001 (***), *p* < 0.01 (**), and *p* < 0.05 (*). The detailed results of the exact *p*-values are shown in Table S3.

## 3. Results

### 3.1. Impact of BPE Exposure on Zebrafish Larvae’s Early Development and Motor Behavior

**Figure 1:**
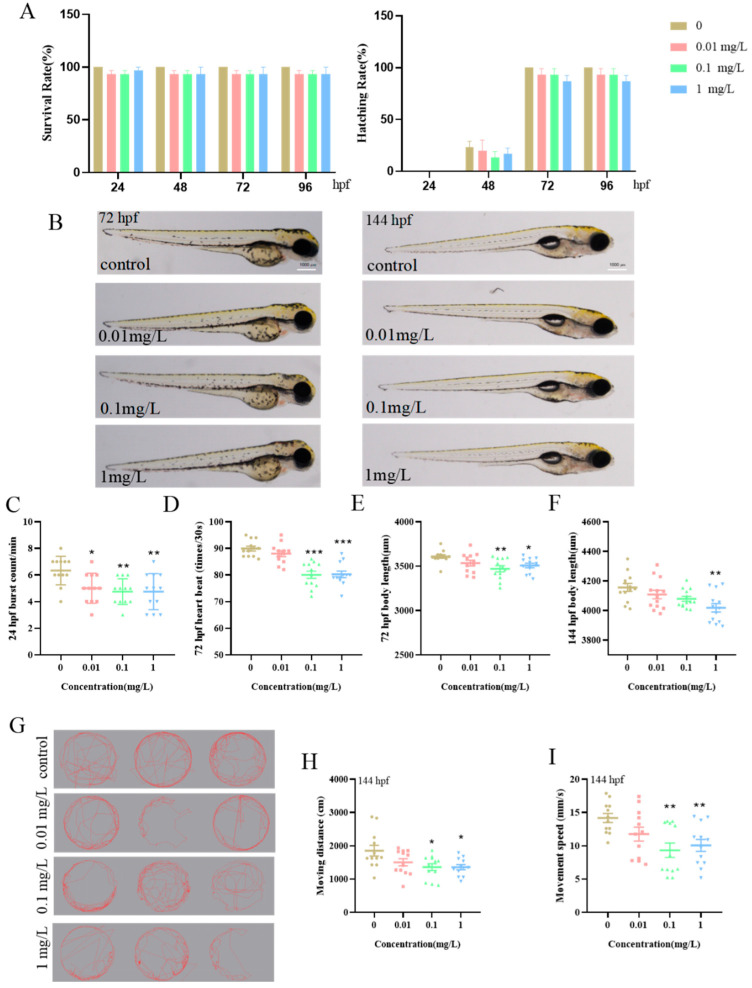
Impact of BPE exposure on zebrafish larval development and motor behavior. (**A**) Larval survival and hatching rates following exposure until 96 hpf. (**B**) Representative morphological images during early development. (**C**) Embryonic tail movement patterns at 24 hpf post-exposure. (**D**) Heart rate and (**E**) body length measurements at 72 hpf. (**F**) Body length, (**G**) motor behavior trajectories, (**H**) moving distance, and (**I**) moving speed of zebrafish larvae at 144 hpf. *p* values: *p* < 0.001 (***), *p* < 0.01 (**), and *p* < 0.05 (*).

BPE may adversely affect early development in zebrafish larvae. Zebrafish embryos were exposed to a concentration gradient of BPE (0.01, 0.1, and 1 mg/L) and monitored continuously from 4 hpf to 144 hpf. Results indicated that at exposure concentrations ≤ 1 mg/L, neither the embryonic survival rate nor the hatch rate showed statistically significant differences between treated groups and the control group (Figure 1A). At 24 hpf, the frequency of spontaneous movements per minute in embryos exposed to BPE significantly decreased, starting from 0.01 mg/L (Figure 1C, *p* < 0.05). By 72 hpf, exposure to 0.1 mg/L and higher concentrations of BPE significantly reduced the heart rate of zebrafish larvae (Figure 1D, *p* < 0.001). The larval body length was shortened at 72 hpf (from 0.1 mg/L) and 144 hpf (from 1 mg/L) following the BPE exposure (Figure 1E,F). Additionally, both the swimming distance and movement speed of larvae exposed to BPE until 144 hpf were affected within the same observation period (Figure 1H,I). Collectively, these results indicate that BPE exposure impacts the early growth, developmental morphology, and motor behavior of zebrafish larvae from embryonic hatching to larval stages.

### 3.2. Effects of Exposure to BPE on Neurodevelopment of Zebrafish Larvae

**Figure 2:**
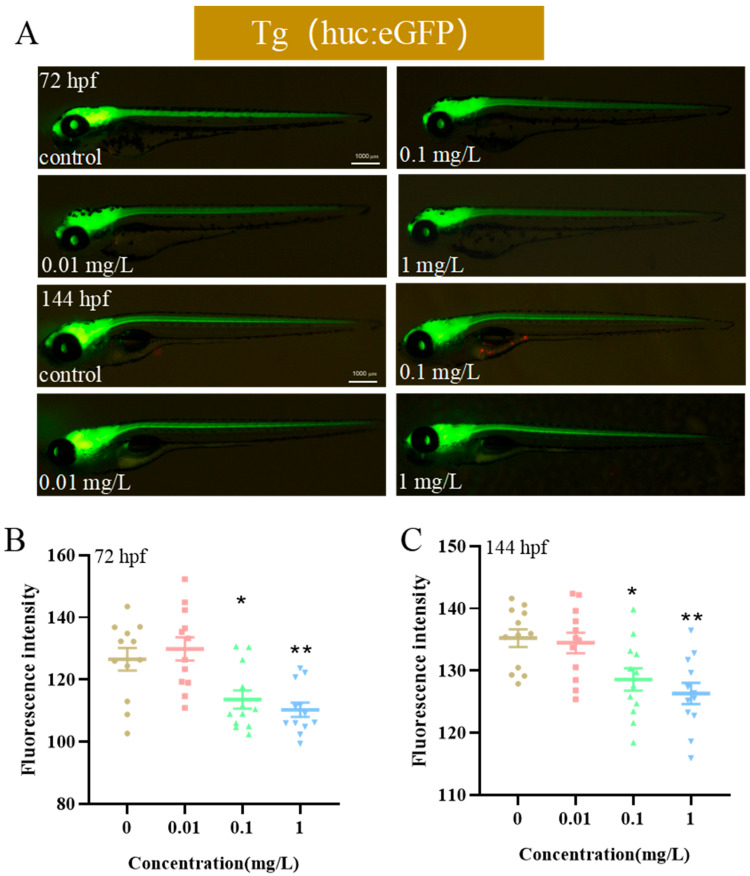
Impact of BPE exposure on zebrafish larval neurodevelopment. (**A**) Representative images of transgenic Tg(huc:eGFP) zebrafish at 72 hpf and 144 hpf post-exposure. (**B**) Fluorescence intensity quantification at 72 hpf and (**C**) 144 hpf. *p* values: *p* < 0.01 (**) and *p* < 0.05 (*).

Motor deficits show strong neurological correlations [26]. To examine BPE impacts on zebrafish neural development, the *Tg(huc:eGFP)* strain, genetically engineered to express a central nervous system (CNS) fluorescence, was exposed to BPE until 144 hpf. Quantitative measurements of neurotoxic effects were conducted via fluorescence stereomicroscopy at 72 hpf and 144 hpf (Figure 2A). The analysis revealed that ≥0.1 mg/L BPE exposure reduced the overall green fluorescence intensity in both cerebral areas and the spinal cord of transgenic zebrafish at these developmental stages (Figure 2B,C, *p* < 0.05). These findings indicate that BPE inhibits neurodevelopment in zebrafish larvae.

### 3.3. Potential Targets for BPE-Induced Neurotoxicity

**Figure 3:**
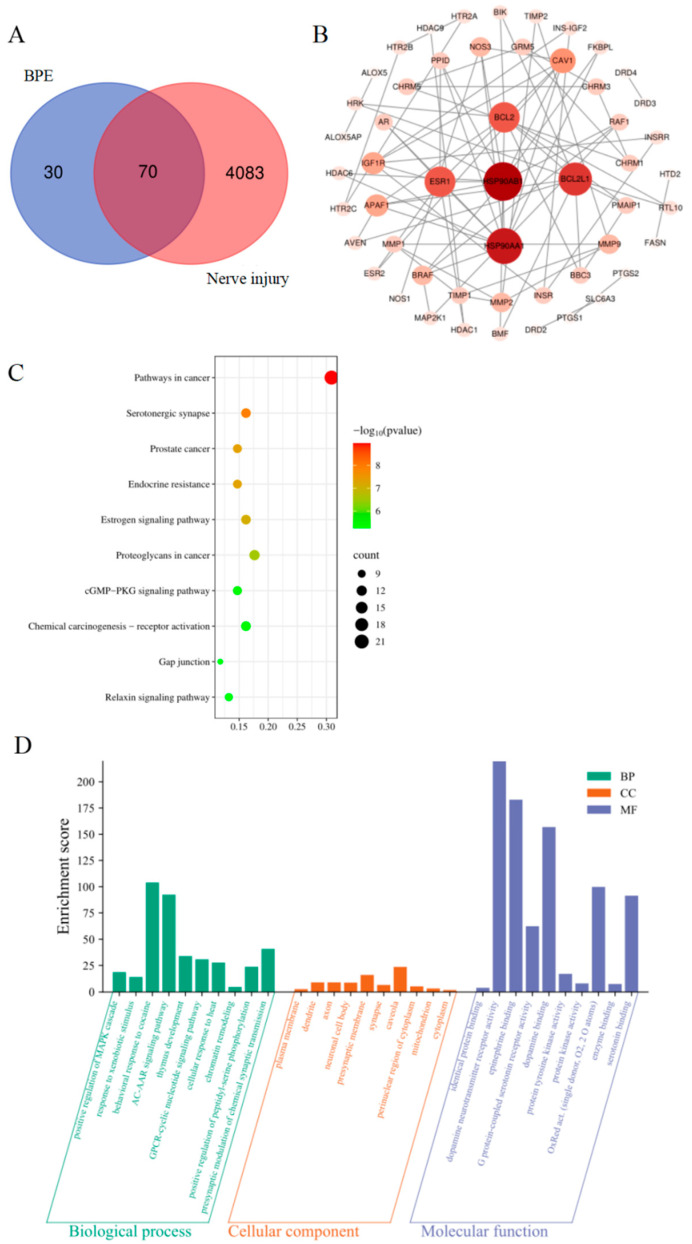
Prediction of BPE toxicity based on network toxicology. (**A**) Venn diagram of BPE toxicity targets and nerve injury targets. (**B**) PPI network of potential targets. (**C**) KEGG enrichment analysis of potential targets. (**D**) GO enrichment analysis of potential targets, including Biological Process (BP), Cellular Component (CC), and Molecular Function (MF).

In this study, 100 potential targets of BPE and 4153 neural damage-related targets were, respectively, screened using the SwissTarget Prediction and GeneCards databases. Following the integration and deduplication of these target sets, a total of 70 overlapping targets were identified, which were considered as potential targets for BPE-induced neurotoxicity (Figure 3A).

### 3.4. Major Interaction Networks and Core Genes of Potential Targets

The STRING database was used to build a protein–protein interaction (PPI) network, which was then exported for an additional examination. Using the Cytoscape 3.10.3 program, the topological properties of network nodes, including the degree and betweenness centrality, were investigated. Concurrently, a visually optimized major PPI network diagram was generated (Figure 3B). The network analysis identified a set of 52 core targets for BPE-induced neurotoxicity, as detailed in Supplementary Table S2. Notably, the top five targets ranked by degree values were the heat shock protein 90 alpha family class B member 1 (HSP90AB1), heat shock protein 90 alpha family class A member 1 (HSP90AA1), B-cell lymphoma 2-like protein 1 (BCL2L1), B-cell lymphoma 2 (BCL2), and Estrogen Receptor 1 (ESR1), with HSP90AB1 exhibiting the highest degree value. Proteins encoded by these genes influence neurogenesis, neuronal migration, survival, and synaptic formation, and the aberrant expression of any of them may lead to structural or functional neural deficits.

### 3.5. Functional Analysis of Targets and Pathway Enrichment Analysis

To elucidate BPE’s potential toxic mechanisms, the Kyoto Encyclopedia of Genes and Genomes (KEGG) pathway analysis of 70 candidate targets was conducted via the DAVID database. A significance bubble chart visually ranked the top 10 enriched pathways by *p*-values (Figure 3C). Concurrently, the Gene Ontology (GO) analysis restricted to Homo sapiens identified 507 significant terms from the same targets: 309 Biological Processes (BPs), 58 Cellular Components (CCs), and 140 Molecular Function (MF) entries. The minimal *p*-value terms (top 10 per category) were visualized in an enrichment diagram (Figure 3D).

The KEGG enrichment analysis revealed significantly enriched pathways, such as cancer signaling (involving aberrant activation/inactivation during tumorigenesis and metastasis), serotonergic synapse, and cGMP/PKG signaling. The GO analysis further indicated a pronounced enrichment in G protein-coupled receptor (GPCR) pathways mediated by cyclic nucleotide second messengers. These mechanistic insights align with observed neurodevelopmental impairment phenotypes.

### 3.6. Molecular Docking of BPE with Core Target Proteins in Neural Damage

**Figure 4:**
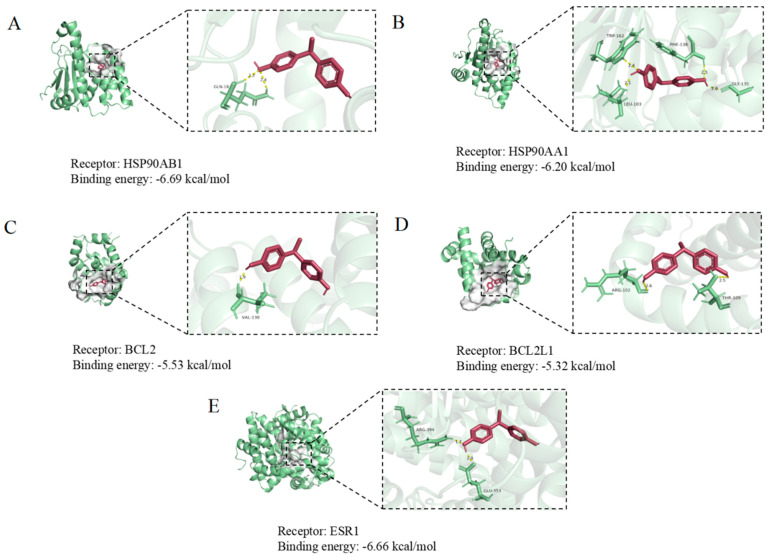
Molecular docking results of BPE with core targets HSP990AB1 (**A**), HSP90AA1 (**B**), BCL2 (**C**), BCL2L1 (**D**), and ESR1 (**E**).

Molecular docking was employed to examine BPE interactions with core target genes. The BPE ligand structure was acquired from PubChem and energy-minimized, while protein receptor crystal structures were downloaded from the Protein Data Bank (PDB). Ligand–receptor docking simulations were executed using AutoDockTools-1.5.6 (Scripps Research). Binding conformations with minimal energy were visualized in PyMOL 2.3.4 and Discovery Studio 2024 (Figure 4). Notably, the core target HSP90AB1 (PDB ID: 6N8Y) demonstrated optimal binding energy (−6.69 kcal/mol), indicating a high binding affinity with BPE. This finding underscores HSP90AB1’s pivotal role in BPE-mediated neurotoxicity mechanisms.

### 3.7. Expression of Neurotoxicity-Related Pathway Genes in Zebrafish Larvae Induced by BPE Exposure

**Figure 5:**
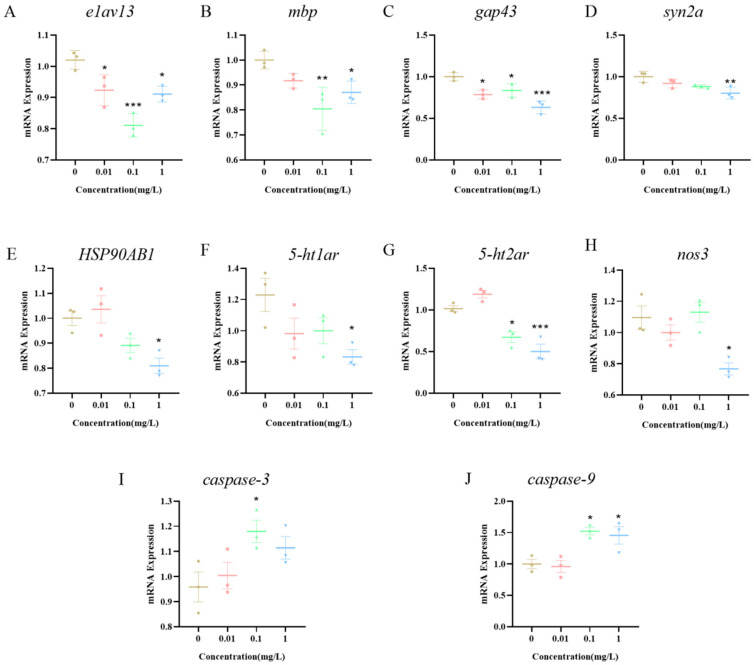
Expression levels of genes related to neurodevelopment (**A**–**E**), serotonergic synapse signaling pathway (**F**,**G**), cGMP/PKG pathway (**H**), and apoptosis (**I**,**J**) in zebrafish larvae exposed to BPE. *p* values: *p* < 0.001 (***), *p* < 0.01 (**), and *p* < 0.05 (*).

To further investigate the potential mechanisms by which BPE affects neurodevelopment in zebrafish larvae, embryos were exposed to BPE until 144 hpf, followed by an RNA extraction and a quantitative analysis of the related gene expression. The results showed that the expression of neurodevelopment-related genes (*elav3*, *mbp*, *gap43*, and *syn2a*) was significantly inhibited (Figure 5A–D). This finding is consistent with the previous results of this study; that is, the exposure to BPE at concentrations ≤ 1 mg/L can inhibit the overall development and central nervous system development of zebrafish larvae. At an exposure concentration of 1 mg/L, the expression of the *HSP90AB1* gene was significantly decreased (Figure 5E, *p* < 0.05), and genes related to serotonergic synaptic signaling (*5-ht1ar*, *5-ht2ar*) and the core gene of the cGMP/PKG pathway (*nos3*) were all significantly inhibited (Figure 5F–H). This indicates that in zebrafish larvae, BPE can downregulate the expression of the *nos3* gene by inhibiting the levels of genes associated with 5-TH receptors. Compared with the control group, expression levels of pro-apoptotic genes (*caspase-3*, *caspase-9*) were significantly elevated, suggesting that BPE induces neuronal apoptosis in zebrafish larvae.

## 4. Discussion

BPE, a typical EDC, is ubiquitously present in aquatic environments, posing significant threats to human health and the ecological balance [2]. However, its neurotoxic effects and underlying mechanisms remain poorly understood. In this study, we employed a series of in silico toxicology prediction tools to screen BPE-induced neurodamage-related targets using the SwissTarget Prediction and GeneCards databases. A protein–protein interaction network of potential targets was constructed via the STRING platform and Cytoscape 3.10.3 software, identifying HSP90AB1 as a core target for BPE-induced neurotoxicity in zebrafish larvae. Furthermore, the toxicological and mechanistic validation via experimental assays and the RT-qPCR provided a comprehensive evidence chain for these findings.

Zebrafish (*Danio rerio*) offer unique advantages for medium-throughput toxicology screening, including a low cost, high fecundity, and rapid development, enabling the fast assessment of compound toxicity and molecular mechanism studies [18,27]. Consequently, zebrafish have emerged as a pivotal model in biomedical research and drug screening. In this study, the BPE exposure significantly reduced the frequency of embryonic tail movements at 24 hpf compared to controls. Additionally, the larval body length was significantly shortened at 72 hpf (from 0.1 mg/L) and 144 hpf (from 1 mg/L), consistent with prior reports that EDCs inhibit growth in zebrafish [28]. Motor behavior, a critical indicator of neurodevelopment, is widely used to evaluate the neurotoxicity of environmental pollutants. The zebrafish larval movement is controlled by spinal neurons, making motor metrics valuable for assessing neurotoxicity induced by environmental exposures [29]. Our results showed that the BPE exposure reduced the swimming distance and speed in larvae, mirroring effects of the EDC BPA on zebrafish motor behavior [30]. This suggests that BPE shares neurobehavioral inhibitory properties with BPA. Collectively, findings from early development and motor behavior assays support the hypothesis that BPE impacts the nervous system of zebrafish larvae.

Aquatic transgenesis constitutes an innovative approach that advances the comprehension of genetic mechanisms and embryogenesis, simultaneously establishing refined integrated models for chemical health risk assessments [31]. Zebrafish models incorporating transgenic technology have been employed to investigate neural development [32]. This methodology involves fusing enhanced the green fluorescent protein (EGFP) with the elavl3 (*huc*) promoter to drive the neuron-specific expression of RNA-binding proteins [33,34]. The expression of the GFP is driven by *huc*, a key gene in the development of central and motor nerves [35]. Also known as elavl3, it is primarily characterized by its specific expression in neuronal cells. The activity of the *huc* promoter can first be detected during the somite stage of zebrafish embryonic development (approximately 10 h), with an initial expression in neural progenitor cells of the forebrain, midbrain, and hindbrain. As the embryonic development proceeds (e.g., from 24 hpf to 48 hpf), its expression gradually expands to the entire nervous system, including differentiated neurons in regions such as the brain, spinal cord, and retina [36]. We employed *Tg(huc:eGFP)* transgenic zebrafish to evaluate BPE effects on larval neurodevelopment. Results showed that the BPE exposure at 72 hpf and 144 hpf reduced the fluorescence intensity. Previous studies using *Tg(huc:eGFP)* demonstrated that bisphenol A and derivatives induce neurotoxicity in zebrafish larvae [37], reducing central nervous system neurogenesis—findings consistent with our results. Notably, *elavl3* is a critical regulator of neuronal development, modulating the neurospecific gene expression via RNA-binding functions. Its specific expression in model organisms like zebrafish makes it an essential molecular tool for studying neurodevelopment, neurotoxicity, and neurological disorders [38]. The *syn2a* gene is closely linked to zebrafish neurodevelopment and synaptic function, with mutations affecting behavior [39]. *Gap43* guides axonal growth and regulates synaptogenesis during the neural differentiation of bone marrow mesenchymal stem cells [40]. *Mbp*, a core gene for central nervous system myelination, is crucial for rapid nerve impulse conduction [41]. The suppressed expression of neurodevelopment-related genes further confirms a positive correlation between BPE-induced motor behavior inhibition and central neural damage in zebrafish larvae. Meanwhile, this study found that there are concentration- and age-dependent differences in the results of the general development and neurodevelopment. The non-monotonic dose–response (NMDR) of bisphenol environmental endocrine disruptors (EDCs) has been widely demonstrated [42]. In this study, a typical biphasic response was observed in the zebrafish model: low doses (0.01–0.1 mg/L) may partially activate neural pathways through estrogen receptors (Esrs) (Figure 3C), producing transient stimulatory effects (such as motor enhancement at 72 hpf); in contrast, high doses (≥1 mg/L) trigger HSP90AB1-mediated cellular stress and caspase-dependent apoptosis (Figure 5), leading to neurological damage and behavioral inhibition. This NMDR may be related to the ability of EDCs to interfere with receptor homeostasis [42]. The specific mechanism behind this phenomenon requires further research.

The serotonergic synapse, a synaptic connection in the central nervous system (CNS) that uses serotonin (5-hydroxytryptamine, 5-HT) as a neurotransmitter, plays a central role in regulating physiological and psychological functions such as mood, sleep, appetite, pain, and cognition [43]. Serotonergic signaling is one of the high-frequency pathways associated with neuroleptic malignant syndrome (NMS), and the neurotransmission at serotonergic synapses is susceptible to interference [44]. Studies have shown that acrylamide exposure significantly upregulates the transcription of serotonergic synapse-related genes, interfering with 5-HT synthesis, transport, and release, disrupting synaptic transmission stability, and inducing neurotoxicity in *Daphnia magna* with resultant motor behavior deficits [45]. The cGMP/PKG signaling pathway, an important second messenger system in vivo, regulates multiple physiological and pathological processes by activating protein kinase G (PKG) via cyclic guanosine monophosphate (cGMP). This pathway is critical for neural signal transduction [46]. In mouse dorsal root ganglion (DRG) neurons, cGMP-dependent protein kinase Iα (cGKIα) induces growth cone collapse, and neurons from cGKIα-deficient mice show an impaired function [47]. In this study, the findings of the network toxicology indicate that serotonergic synapses and the cGMP/PKG signaling pathway are anticipated to play a pivotal role in the neurotoxic mechanism of BPE. This discovery potentially correlates with the known neurotoxic effects of bisphenols. The existing research has confirmed that bisphenols can interfere with neural development processes by binding to estrogen receptors [48]. Building on this, the present study further reveals that the estrogen signaling pathway and dopaminergic signaling pathway are significantly enriched, and their interaction may be involved in the regulation of neurotoxicity: on the one hand, the dopaminergic pathway can indirectly affect the synthesis and secretion of estrogen by regulating the hypothalamic–pituitary–gonadal (HPG) axis; on the other hand, estrogen can also inversely regulate key components of the dopaminergic pathway, thereby exerting developmental toxicity on zebrafish embryos and larvae [49]. This interaction suggests that the two may indirectly affect the growth and development of zebrafish larvae through a synergistic effect. In addition, the direct interaction between estrogen receptors and 5-HT receptors in neurons has also been confirmed to be involved in neural regulation. For example, estrogen receptors can reduce the sensitivity to 5-HT by inhibiting the transcriptional activity of the 5-HT1A receptor, leading to imbalances in postsynaptic signal transmission [50]; similarly, the dopaminergic pathway can inhibit 5-HT1A receptor-mediated neural signals in hippocampal slice models. Another study shows that the neuronal mortality rate in 5-HT1A receptor knockout mice increases by 50% [51], which is consistent with the results of the present study, which show that the expression levels of 5-HT receptor-related genes (*5-ht1ar* and *5-ht2ar*) are inhibited. This suggests that BPE may interfere with the function of the 5-HT system by affecting the estrogen signaling pathway and dopaminergic pathway, thereby inducing neurotoxicity in zebrafish larvae and inhibiting their motor behavior. This result also reveals the potential complexity of the toxicity mechanism of such pollutant exposure, and the specific details of its action still need to be further explored through more experiments.

The core target gene *HSP90AB1*, a key member of the heat shock protein family, plays multifaceted roles in cells and is intricately linked to various physiological and pathological processes. In cancer, it is often upregulated, promoting tumor growth, proliferation, and metastasis by stabilizing oncogenic proteins [52]. Studies have shown that the high expression of *HSP90AB1* in neural stem cells (NSCs) activates the NF-κB/p65 signaling pathway, promoting NSC apoptosis [53]. Molecular docking, a widely used in silico screening method, aims to predict the binding conformation of small-molecule ligands to targets [54]. It not only simulates binding sites and interaction types between compounds and receptors but also evaluates the complex stability, providing evidence for unraveling toxic mechanisms of compounds in vivo [55]. This technique has been extensively applied to predict and analyze potential mechanisms of endocrine disruptor-induced toxicity [56,57,58]. Findings from this study show that BPE binds to the *HSP90AB1* protein with a binding energy of −6.69 kcal/mol, forming a relatively stable complex. This indicates an interaction of considerable strength, with subsequent neural effects validating our previous predictions. While in silico toxicology and molecular docking play pivotal roles in toxicology research and drug development, challenges such as data dependency, mechanistic inference limitations, and computational resource constraints highlight the need for experimental validation to enhance result reliability.

This study found that the *HSP90AB1* gene expression was significantly decreased at 1 mg/L, indicating functional inhibition. Previous studies have shown that HSP90 participates in neurotransmitter release [59]. Concomitantly, the gene expression of *5-ht1ar* and *5-ht2ar* was significantly suppressed, suggesting that BPE inhibits the release and transmission of serotonin (5-HT) in zebrafish larvae, thereby affecting neuronal excitability and synaptic plasticity. The reduced *HSP90AB1* expression and aberrant 5-HT release impacted the cGMP/PKG signaling pathway. Nitric oxide (NO), a paracrine signaling molecule, increases cGMP levels by activating soluble guanylate cyclase (sGC), thereby activating PKG [60]. In our study, BPE exposure significantly suppressed the *nos3* (endothelial nitric oxide synthase, eNOS) gene expression (as measured by RT-qPCR). This downregulation of the nos3 expression is consistent with a potential reduction in NO production, based on the established role of eNOS as the enzyme catalyzing NO synthesis. Importantly, the existing literature demonstrates that bisphenol A can induce eNOS uncoupling, shifting its enzymatic function from producing NO to generating superoxide anions (O_2_^−^), thereby effectively inhibiting its normal signaling role [61]. This uncoupling mechanism represents a direct functional perturbation of eNOS activity. Consequently, the observed suppression of the nos3 expression, combined with the well-documented potential for bisphenol-induced eNOS uncoupling, strongly suggests an impairment of the NOS3 activity in BPE-exposed larvae. The loss of NOS3 delays cortical neuronal migration in mice, impairing neurodevelopment [62]. Consistently, BPE exposure significantly suppressed the *NOS3* expression, reducing NO production and destabilizing the NOS3/PKG axis, demonstrating adverse effects on cGMP/PKG signaling and neurodevelopment in zebrafish larvae. Our findings of a suppressed *nos3* gene expression and the predicted disruption of downstream signaling (via network toxicology and molecular docking) collectively demonstrate adverse effects on the cGMP/PKG pathway and neurodevelopment in zebrafish larvae. *Caspase-9*, a cysteine protease family member, is essential for apoptosis during central nervous system development [63], while *caspase-3* plays a central role in programmed cell death [64]. BPE exposure significantly upregulated *caspase-9* and *caspase-3* expression, indicating excessive neuronal cell death. In neurodegenerative diseases, cGMP/PKG pathway inhibition activates the *caspase-9/caspase-3* cascade to induce neuronal death [65]. In summary, these results indicate that BPE induces neurotoxicity in zebrafish larvae by inhibiting the expression of genes related to the serotonin synaptic pathway, disrupting cGMP/PKG, and promoting apoptosis, ultimately leading to deficits in motor behavior.

## 5. Conclusions

This study used zebrafish as a model and integrated in silico toxicology, molecular docking, and RT-qPCR techniques to reveal that BPE can induce neurodevelopmental disorders and motor deficits in larvae through pathways such as inhibiting the expression of *5-HT* receptors, reducing signal transduction efficiency, disrupting the cGMP/PKG pathway (with the mechanism being the reduction in NOS3/PKG stability), and promoting apoptosis. The above findings provide key mechanistic data and environmental risk assessment evidence for evaluating the neurotoxicity of BPE to aquatic organisms, fill relevant research gaps, and offer new perspectives for the study of pollutant-mediated neurodevelopmental disorders. Future research needs to conduct long-term low-dose exposure experiments to systematically assess its chronic effects.

## Data Availability

The raw data supporting the conclusions of this article will be made available by the authors upon request.

## Supplementary Materials

The following supporting information can be downloaded at https://www.mdpi.com/article/10.3390/biology14080992/s1: Table S1: Candidate targets screened from the PPI network; Table S2: The primer sequences for QPCR; Figure S1: guidelines for the care and use of laboratory animals; and Table S3: The detailed information of statistical tests between the exposure groups and control.
