# Supplementary material for: Bisphenol E Neurotoxicity in Zebrafish Larvae: Effects and Underlying Mechanisms
Source: Biology (Basel). 2025 Aug 4;14(8):992. doi: 10.3390/biology14080992 (PMC12383493; doi:10.3390/biology14080992)
Supplement: Supplementary file 1 [file biology-14-00992-s001.zip › biology-3734082-supplementary.pdf]

Supplementary materials

Supplementary Table 1

Candidate targets screened from the PPI network.

Name	Degree	Closeness centrality	Betweenness centrality	Topological coefficient
HSP90AB1	11	0.5087719298245614	0.36494252873563215	0.23923444976076555
HSP90AA1	10	0.5	0.28160919540229895	0.25263157894736843
BCL2L1	9	0.3625	0.18842364532019704	0.29292929292929293
BCL2	8	0.35802469135802467	0.1724137931034483	0.3181818181818182
ESR1	8	0.4328358208955224	0.2697044334975369	0.3014705882352941
CAV1	6	0.42028985507246375	0.13916256157635468	0.37037037037037035
APAF1	5	0.4393939393939394	0.4445812807881773	0.4
IGF1R	5	0.3372093023255814	0.1933497536945813	0.35
BRAF	4	0.35802469135802467	0.04597701149425286 6	0.5416666666666666
NOS3	4	0.3815789473684211	0.06896551724137931	0.5192307692307693
MMP2	4	1	0.16666666666666666	0.75
MMP9	4	1	0.16666666666666666	0.75
AR	3	0.3815789473684211	0	0.6904761904761905
BBC3	3	0.27358490566037735	0	0.7407407407407407
PMAIP1	3	0.27358490566037735	0	0.7407407407407407
RAF1	3	0.3536585365853659	0.02175697865353038	0.4722222222222222
CHRM1	3	1	0	1
CHRM3	3	1	0	1
GRM5	3	1	0	1
CHRM5	3	1	0	1
PPID	3	0.3815789473684211	0	0.6904761904761905
INSR	3	0.2589285714285714	0.0012315270935960591	0.6
MMP1	3	0.8	0	0.9166666666666666
TIMP1	3	0.8	0	0.9166666666666666
AVEN	2	0.3333333333333333	0	0.6363636363636364
BIK	2	0.27102803738317754	0	0.9444444444444444
HRK	2	0.27102803738317754	0	0.9444444444444444
BMF	2	0.27102803738317754	0	0.9444444444444444
RTL10	2	0.27102803738317754	0	0.9444444444444444
MAP2K1	2	0.26851851851851855	0	0.875
ESR2	2	0.31521739130434784	0	0.7777777777777778
HDAC1	2	0.3118279569892473	0.06896551724137931	0.5
FKBPL	2	0.34523809523809523	0	0.9545454545454546
HDAC6	2	0.34523809523809523	0	0.9545454545454546
HTR2A	2	1	0	1
HTR2B	2	1	0	1
HTR2C	2	1	0	1

INSRR	2	0.25663716814159293	0	0.8
INS-IGF2	2	0.25663716814159293	0	0.8
TIMP2	2	0.6666666666666666	0	1
ALOX5	1	1	0	0
ALOX5AP	1	1	0	0
DRD2	1	1	0	0
SLC6A3	1	1	0	0
DRD3	1	1	0	0
DRD4	1	1	0	0
FASN	1	1	0	0
HTD2	1	1	0	0
HDAC9	1	0.2396694214876033	0	0
NOS1	1	0.27884615384615385	0	0
PTGS1	1	1	0	0
PTGS2	1	1	0	0

Supplementary Table 2 The primer sequences for QPCR.

Gene	Forward (5'-3')	Reverse (5'-3')
β-actin	GGAGATTACTGCCCTGGCTCCTA	GACTCATCGTACTCCTGCTTGCTG
elavl3	AGACAAGATCACAGGCCAGAGCTT	TGGTCTGCAGTTTGAGACCGTTGA
mbp	AATCAGCAGGTTCTTCGGAGGAGA	AAGAAATGCACGACAGGGTTGACG
syn2a	GTGACCATGCCAGCATTTC	TGGTTCTCCACTTTCACCTT
gap43	TGCTGCATCAGAAGAACTAA	CCTCCGGTTTGATTCCATC
HSP90AB1-F	ACAACATCAAGCTGTACGTC	GGATGAAGTTCAGATACTCTGG
5-HT1AR-F	TGGCTCATTGGCTTTCTCATCTC	AGCAGCAGCGGAATATAGAAAGC
5-HT2AR	TATGCTGCTGGGTTTCCTTGTC	GGAGAAGAGCACATCCAGGTAAATC
NOS3	GGGTCCTGTGTATGGATGAG	TTATAACTCTTGTGCTGTTCCG
Caspase-3	CCGCTGCCCATCACTA	ATCCTTTCACGACCATCT
Caspase-9	AAATACATAGCAAGGCAACC	CACAGGGAATCAAGAAAGG

Figure S1

动物实验伦理审查同意书
Affidavit of Approval of Animal Ethical and Welfare

申请编号<br>Application No:	20250401	批准编号<br>Approval No:	IACUC-20250401

本《动物实验方案》经过实验动物伦理委员会审核，符合动物保护、动物福利和伦理原则，符合国家实验动物福利伦理的相关规定。方案的相关信息如下：

The animal use protocol listed below has been reviewed and approved by the Animal Ethical and Welfare Committee (AEWC).

<table>
<tr><td>实验名称
Protocol Title</td><td colspan="5">双酚 E 对斑马鱼幼鱼神经系统的影响及机制探究</td></tr>
<tr><td rowspan="2">申请人姓名
Applicant</td><td>陈健楠</td><td rowspan="2">职称/学位
Title/Degree</td><td>博士</td><td rowspan="2">邮箱
Email</td><td rowspan="2">cjn.njnu@foxmail.com</td></tr>
<tr><td>Jiannan Chen</td><td>Doctor</td></tr>
<tr><td rowspan="2">实验负责人
Principle Investigator</td><td>郭志刚</td><td rowspan="2">职称/学位
Title/Degree</td><td>教授</td><td rowspan="2">邮箱
Email</td><td rowspan="2">08278@njnu.edu.cn</td></tr>
<tr><td>Zhigang Guo</td><td>Professor</td></tr>
<tr><td rowspan="2">院系（部门）
Department</td><td colspan="3">生命科学学院</td><td rowspan="2">申请日期
Application Date</td><td rowspan="2">2025.04.01</td></tr>
<tr><td colspan="3">School of Life Sciences</td></tr>
<tr><td rowspan="2">动物种系
Species or Strains</td><td colspan="3">AB 野生型斑马鱼，转基因斑马鱼 Tg（huc：eGFP）和转基因斑马鱼 Tg（hb9：eGFP）</td><td rowspan="2">数量
Quantity</td><td rowspan="2">100</td></tr>
<tr><td colspan="3">Zebrafish (Danio rerio), including AB wild-type, Tg(huc:eGFP), and Tg(hb9:eGFP) transgenic lines.</td></tr>
<tr><td>拟实验时间
Period of Protocol</td><td>2025.04.01-2025.06.05</td><td colspan="2">实验动物使用许可证
Number of Animal Use Permit</td><td colspan="2"></td></tr>
<tr><td>审核意见
Results of Inspection</td><td colspan="5">☑ 符合动物福利伦理要求，可以进行实验。 Agree
☐ 调整方案后，可以进行实验。 Agree after modify</td></tr>
</table>

南京师范大学实验动物福利伦理审查委员会

Animal Ethical and Welfare Committee of NNU

主席(Chairman):

日期(Date): 2025.04.01

Supplementary Table 3 The detailed information of statistical tests between the exposure groups and control

	Concentration (mg/L)	*p*-value
24 hpf burst count/min	0 vs. 0.01	0.0173
	0 vs. 0.1	0.0040
	0 vs. 1	0.0040
72 hpf heart beat (times/30s)	0 vs. 0.01	0.5137
	0 vs. 0.1	＜0.0001
	0 vs. 1	＜0.0001
72 hpf body length(μm)	0 vs. 0.01	0.1734
	0 vs. 0.1	0.0034
	0 vs. 1	0.0401
144 hpf body length(μm)	0 vs. 0.01	0.4179
	0 vs. 0.1	0.0994
	0 vs. 1	0.0012
Moving distance (mm)	0 vs. 0.01	0.0986
	0 vs. 0.1	0.0120
	0 vs. 1	0.0113
Movement speed (mm/s)	0 vs. 0.01	0.1808
	0 vs. 0.1	0.0020
	0 vs. 1	0.0094
72 hpf fluorescence intensity	0 vs. 0.01	0.7999
	0 vs. 0.1	0.0179
	0 vs. 1	0.0024
144 hpf fluorescence intensity	0 vs. 0.01	0.9767
	0 vs. 0.1	0.0174
	0 vs. 1	0.0011
gene		
elavl3	0 vs. 0.01	0.0295
	0 vs. 0.1	0.0003
	0 vs. 1	0.0161
mbp	0 vs. 0.01	0.2142
	0 vs. 0.1	0.0055
	0 vs. 1	0.0455
gap43	0 vs. 0.01	0.0115
	0 vs. 0.1	0.0412
	0 vs. 1	0.0004
syn2a	0 vs. 0.01	0.2570
	0 vs. 0.1	0.0754
	0 vs. 1	0.0064
HSP90AB1	0 vs. 0.01	0.8431
	0 vs. 0.1	0.1677
	0 vs. 1	0.0177

5-ht1ar	0 vs. 0.01	0.1774
	0 vs. 0.1	0.2203
	0 vs. 1	0.0296
5-ht2ar	0 vs. 0.01	0.1839
	0 vs. 0.1	0.0104
	0 vs. 1	0.0009
nos3	0 vs. 0.01	0.5452
	0 vs. 0.1	0.9529
	0 vs. 1	0.0102
caspase-3	0 vs. 0.01	0.8592
	0 vs. 0.1	0.0383
	0 vs. 1	0.1453
caspase-9	0 vs. 0.01	0.9840
	0 vs. 0.1	0.0134
	0 vs. 1	0.0269
